# *Enterococcus faecalis* from Healthy Infants Modulates Inflammation through MAPK Signaling Pathways

**DOI:** 10.1371/journal.pone.0097523

**Published:** 2014-05-15

**Authors:** Shugui Wang, Martin Lloyd Hibberd, Sven Pettersson, Yuan Kun Lee

**Affiliations:** 1 Department of Microbiology, National University of Singapore, Singapore, Singapore; 2 Division of Cellular and Molecular Research, National Cancer Centre Singapore, Singapore, Singapore; 3 Infectious Diseases, Genome Institute of Singapore, Singapore, Singapore; 4 Microbiology and Tumor Biology Center, Karolinska Institute, Stockholm, Sweden; Singapore Immunology Network, Agency for Science, Technology and Research (A*STAR), Singapore

## Abstract

Colonizing commensal bacteria after birth are required for the proper development of the gastrointestinal tract. It is believed that bacterial colonization pattern in neonatal gut affects gut barrier function and immune system maturation. Studies on the development of faecal microbiota in infants showed that the neonatal gut was first colonized with enterococci followed by other microbiota such as *Bifidobacterium*. Other studies showed that babies who developed allergy were less often colonized with *Enterococcus* during the first month of life as compared to healthy infants. Many studies have been conducted to elucidate how bifidobacteria or lactobacilli, some of which are considered probiotic, regulate infant gut immunity. However, fewer studies have been focused on enterococi. In our study, we demonstrate that *E. faecalis*, isolated from healthy newborns, suppress inflammatory responses activated *in vivo* and *in vitro*. We found *E. faecalis* attenuates proinflammatory cytokine secretions, especially IL-8, through JNK and p38 signaling pathways. This finding shed light on how the first colonizer, *E.faecalis*, regulates inflammatory responses in the host.

## Introduction

Significant controversy exists over the role of Enterococcus, and more specifically *E.faecalis*, on health. Whereas, in clinical settings with immune-compromised patients, *E.faecalis* can be considered an opportunistic pathogen [1], it has also been shown to impart beneficial effects to health. A recent *in vitro* study demonstrated that *E. faecalis* was inhibitory to *C. jejuni* MB 4185 infection under simulated broiler caecal condition [2]. An *E.faecalis* isolated from a healthy adult showed the highest probiotic activity when compared with over 70 other lactic acid bacteria (LAB) isolates, including lactobacilli and bifidobacteria [3]. These contrasting roles suggest an interplay between bacteria and human host that is context-dependent and likely dynamic over time.

Prior to birth, the gut is sterile and bacteria start to colonize after birth. The neonatal gut, with a naïve but competent immune system, represents a valuable context for determining the role of particular bacteria in health. Proper development of the gastrointestinal tract requires timely colonization after birth [4]. Study showed microbiota acquisition in infancy is likely a determinant of early immune programming, subsequent infection, and allergy risk [5]. Among the first wave of microorganisms detected in the stool of infants, enterococci are commonly found on the first day of life [6], [7]. They gradually decrease with concurrent increases in *bifidobacteria* that appear within 2–3 days in breast fed infants [8]. Babies who developed allergies were less often colonized with *Enterococcus* during the first month of life as compared to healthy infants [9]. This implies that *Enterococcus* could have major impact on intestinal immune development in the very early stage of life.

Several factors, such as mode of delivery and gestational age, are known to influence the composition of microbial flora in early stage of life. Preterm infants and infants delivered via cesarean section display a delayed intestinal colonization with smaller species variability and a higher occurrence of potentially pathogenic microorganism [10], [11]. Pre-term births are far more likely to suffer necrotizing enterocolitis (NEC) than are births at term [12]. Interestingly, infants who developed NEC carry less *E.faecalis* [13]. Previous studies have shown that serum concentrations of IL-8 were elevated in severe cases of NEC from its onset through the first 24 hours [14]. IL-8 is a chemokine that stimulates migration of neutrophils from intravascular to interstitial sites and can directly activate neutrophils and regulate the expression of neutrophil adhesion molecules [15]–[17]. Thus, IL-8 plays an important role in infant infections.

It is also known that cell-wall components from Gram-negative such as lipopolysaccarides as well as host-derived cytokines such as IL-1β and TNF-α, increase IL-8 secretion from IECs through the activation of mitogen activated protein kinase (MAPK) [18], [19]. At least three groups of MAPKs have been identified. These include the extracellular signal-regulated kinases (ERKs), the c-JUN NH_2_-terminal kinases (JNKs) and p38. P38 was reported to stabilize IL-8 mRNA and its level was increased in the muscularis propria of colonic tissue both in DSS colitis mice and patients with inflammatory bowel disease (IBD) [20]. A p38 inhibitor suppressed inflammation in DSS-induced colitis model by reducing mucosal IL-1β and TNF-α levels [21]. Inhibition of JNK activation correlated with suppression of IL-1β-induced IL-8 secretion in IECs [22]. Study showed activation of the ERK signaling pathway in response to TNF-α in HT-29 cells leads to increased expression of IL-8 [23].

Extensive studies have been performed to determine how lactobacilli and bifidobacteria regulate infant gut immunity [24], [25]. However, few studies have focused on *E.faecalis*, which is the first colonizer in the human GI tract [6], [7]. Here, we demonstrate that *E. faecalis*, isolated from newborns, can suppress pathogen-mediated inflammatory responses in human IECs as well as DSS-induced inflammation in mice model. *E. faecalis* attenuates proinflammatory cytokine secretions, especially IL-8, via distinct pathways. These bacteria suppress JNK and p38 as well as disrupt c-JUN-regulated inflammatory responses. These findings shed lights on functions of the first colonizer, *E.faecalis*, in infant gut protection.

## Results

### Isolation and identification of bacteria from infants' gut

**Figure 1:**
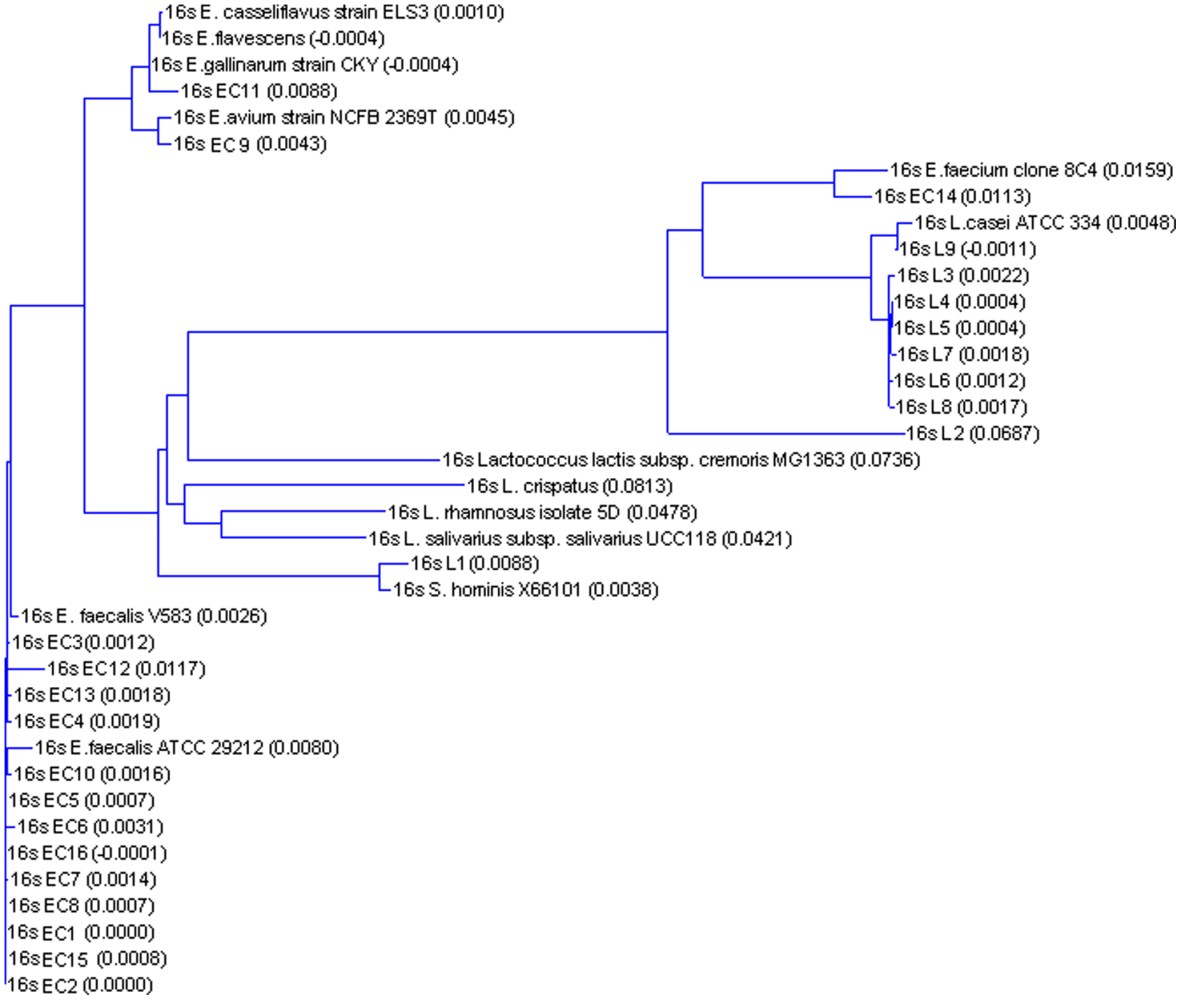
Phylogeny tree of 25 bacterial strains isolated from new born infants according to their 16sRNA sequences.

In order to characterize early postnatal gut LABs, we collected feces from 16 healthy infants aged 3 days and 1 month from Indonesia. A total of 25 isolates were expanded and confirmed as LABs based on lactic acid production, a rod or coccal shape, and Gram positivity. Based on their carbohydrate fermentation patterns, nine strains were categorized as *Lactobacillus* and 16 strains as *Enterococcus*. 16S rDNA sequence analysis showed that 8/9 of the lactobacilli were *Lactobacillus casei* and 13/16 of the enterococci were *Enterococcus faecalis* (Table 1). Thus, we found a restricted diversity of early colonizing species in infants with *L.casei* and *E. faecalis* being the prominent early colonizers. A phylogenetic tree, plotted based on a sequence distance method, is provided (Figure 1). From the phylogenetic tree, we could see some enterococcus were closer to lactobacillus than the rest enterococcus.

**Table 1:** Identification of 25 isolates from infants.

Bacterial names	Bacterial source (Infants Age)	Strain ID obtained from 16S rDNA sequencing
L1	3day	*Staphylococcus hominis*
L2	3day	*Lactobacillus casei*
L5	3day	*Lactobacillus casei*
L6	3day	*Lactobacillus casei*
EC3	3day	*Enterococcus faecalis*
EC6	3day	*Enterococcus faecalis*
EC9	3day	*Enterococcus avium*
EC14	3day	*Enterococcus faecium*
EC15	3day	*Enterococcus faecalis*
EC16	3day	*Enterococcus faecalis*
L3	1month	*Lactobacillus casei*
L4	1 month	*Lactobacillus casei*
L7	1month	*Lactobacillus casei*
L8	1month	*Lactobacillus casei*
L9	1month	*Lactobacillus casei*
EC1	1month	*Enterococcus faecalis*
EC2	1month	*Enterococcus faecalis*
EC4	1month	*Enterococcus faecalis*
EC5	1month	*Enterococcus faecalis*
EC7	1month	*Enterococcus faecalis*
EC8	1month	*Enterococcus faecalis*
EC10	1month	*Enterococcus faecalis*
EC11	1 month	*Enterococcus gallinarum*
EC12	1 month	*Enterococcus faecalis*
EC13	1 month	*Enterococcus faecalis*

### *Enterococcus faecalis* suppress intestinal IL-8 secretion

**Figure 2:**
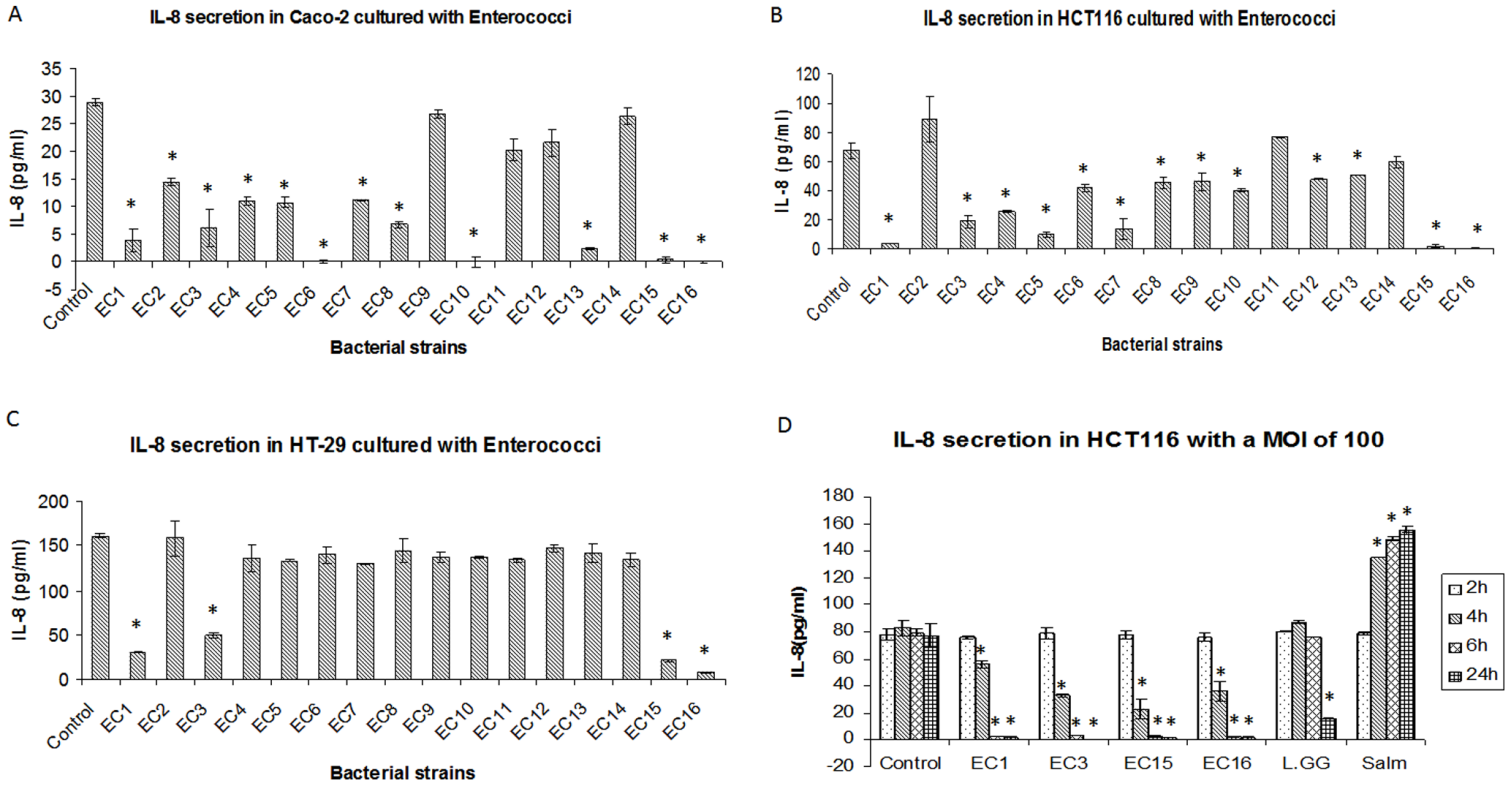
IL-8 secretion in IECs. IL-8 secretions in Caco-2 (A), HCT116 (B) and HT-29 (C) with the treatment of *Enterococcus*. A total number of 10^7^ cfu/ml bacteria were added into the cells for 6 h. Supernatants were harvested for cytokine assay as described in Materials and Methods. (D) HCT116 cells were co-cultured with *E. faecalis* (EC1, EC3, EC15, EC16) and *L. rhamnosus GG* (L.GG) and *S. typhimurium* (Salm) with a multiplicity of infection (MOI) of 100 for 2 h, 4 h, 6 h and 24 hours. Three independent experiments were compiled to produce the data shown. Data were expressed as mean value ±SD. Student's T-test was used for statistical analysis as described in Materials and Methods. * p<0.05.

We then examined how these early colonizing bacteria can impact intestinal immunity and epithelial cell signaling. Potential anti-inflammatory effects of infant-isolated enterococci and lactobacilli were investigated by co-culturing them with colorectal cancer-derived IECs (Caco-2, HT29 and HCT116). Supernatants were harvested for IL-8 analysis as a marker for gut inflammation [26]. Although some lactobacilli suppressed IL-8 secretion in Caco-2 cells, none of the *Lactobacillus* isolates significantly attenuated IL-8 secretion in all the three cell lines (Figure S1A–C). In contrast, the majority of *Enterococcus* isolates suppressed IL-8 production in Caco-2 and HCT116 cells (Figure 2A, B). Strikingly, four strains, namely EC1, EC3, EC15 and EC16, suppressed IL-8 secretion in all three lines (Figure 2 A–C and the reduction of IL-8 levels was not due to apoptosis induced by *E.faecalis* (Figure S1D, E). We then tested the kinetics of IL-8 suppression by these four strains in HCT116 cells (Figure 2D). For each of these isolates, the degree of suppression of IL-8 was dependent on bacterial multiplicity of infection (MOI). *E. faecalis* isolates suppressed IL-8 secretion from 4h at a MOI of 100. However, the same level of suppression was observed much earlier at a MOI of 1000. In contrast, this rapid and robust suppression of IL-8 was not seen with commercial probiotic strains, *L. rhamnosus GG* (L.gg) (Figure 2D). *Salmonella typhimurium* (Salm), a known pathogen and stimulator of IL-8 [27], activated IL-8 secretion after 2 h (Figure 2D). These data suggest that early colonizing *E.faecalis* have potent anti-inflammatory effects as assessed using IL-8 expression as a marker.

### Active *E. faecalis* physiology is not critical for suppression of intestinal inflammation

**Figure 3:**
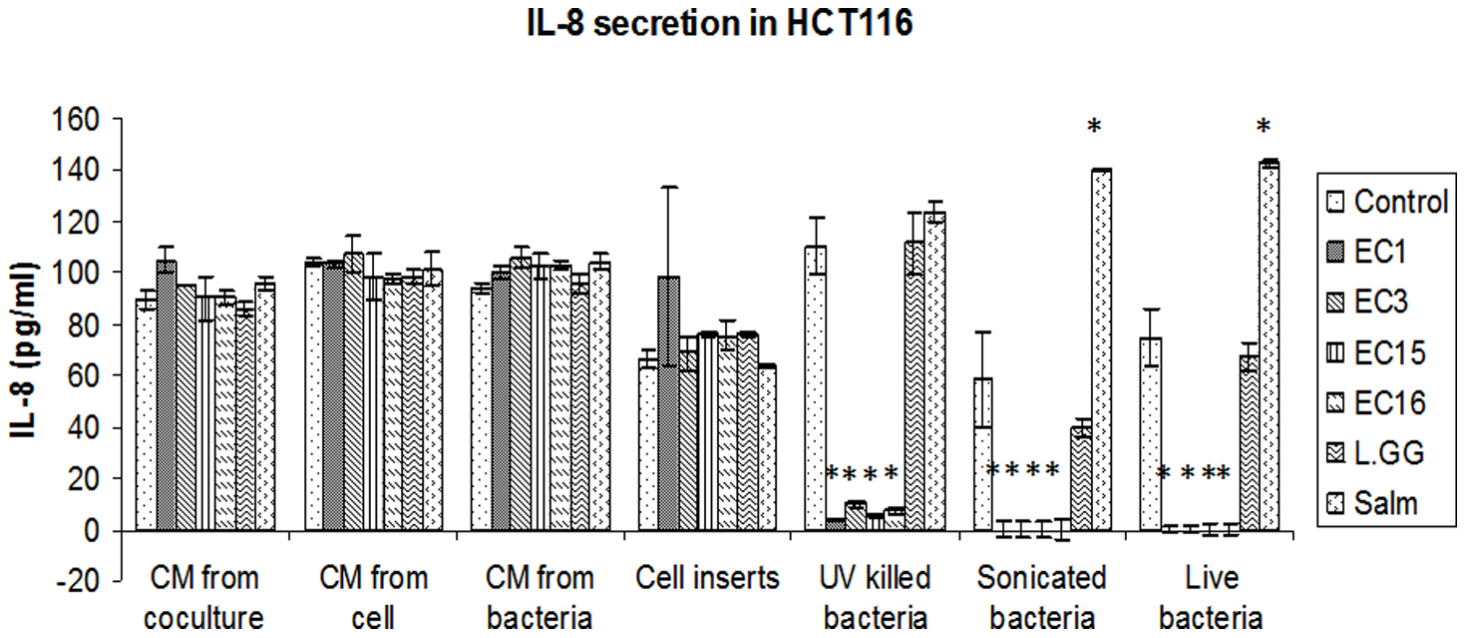
IL-8 secretions in HCT116 cells. IL-8 production in HCT116 with the treatment of different conditional medium (CM) (from coculture supernatant, from bacteria and from cell supernatant), cell inserts, UV killed and sonicated bacteria. Whole live bacteria were used as control as described in Materials and Methods. Three independent experiments were done and data were expressed as mean value ±SD. Student's T-test was used for statistical analysis as described in Materials and Methods. * p<0.05.

We then investigated what aspects of *E. faecalis* may cause reduced IL-8 secretion in IECs. In order to test if intact bacterial physiology was critical for IL-8 suppression, we exposed HCT116 cells to live bacteria or bacteria that had been killed by ultraviolet exposure (UV) or by physical disruption through sonication immediately before use (Figure 3). In each case we found that IL-8 suppression remained intact, indicating that active bacterial physiology was not critical for this activity. We then tested whether physical contact between bacterial membranes and epithelial cells was important. Physically separating live bacteria from the epithelial cells, using a semi-permeable cell culture insert, greatly relieved the IL-8 suppression (Figure 3), indicating that physical contact between the bacteria and epithelial cells was important for this activity. Furthermore, bacteria-conditioned media from bacteria alone or from mammalian cell cocultures resulted in no apparent IL-8 suppression (Figure 3), suggesting that epithelial cells are responding to poorly soluble factor(s), likely present on the exterior cell wall of *E. faecalis*.

### *E. faecalis* suppresses IL-8 production induced by IL-1β, TNF-α and *S. typhimurium*

**Figure 4:**
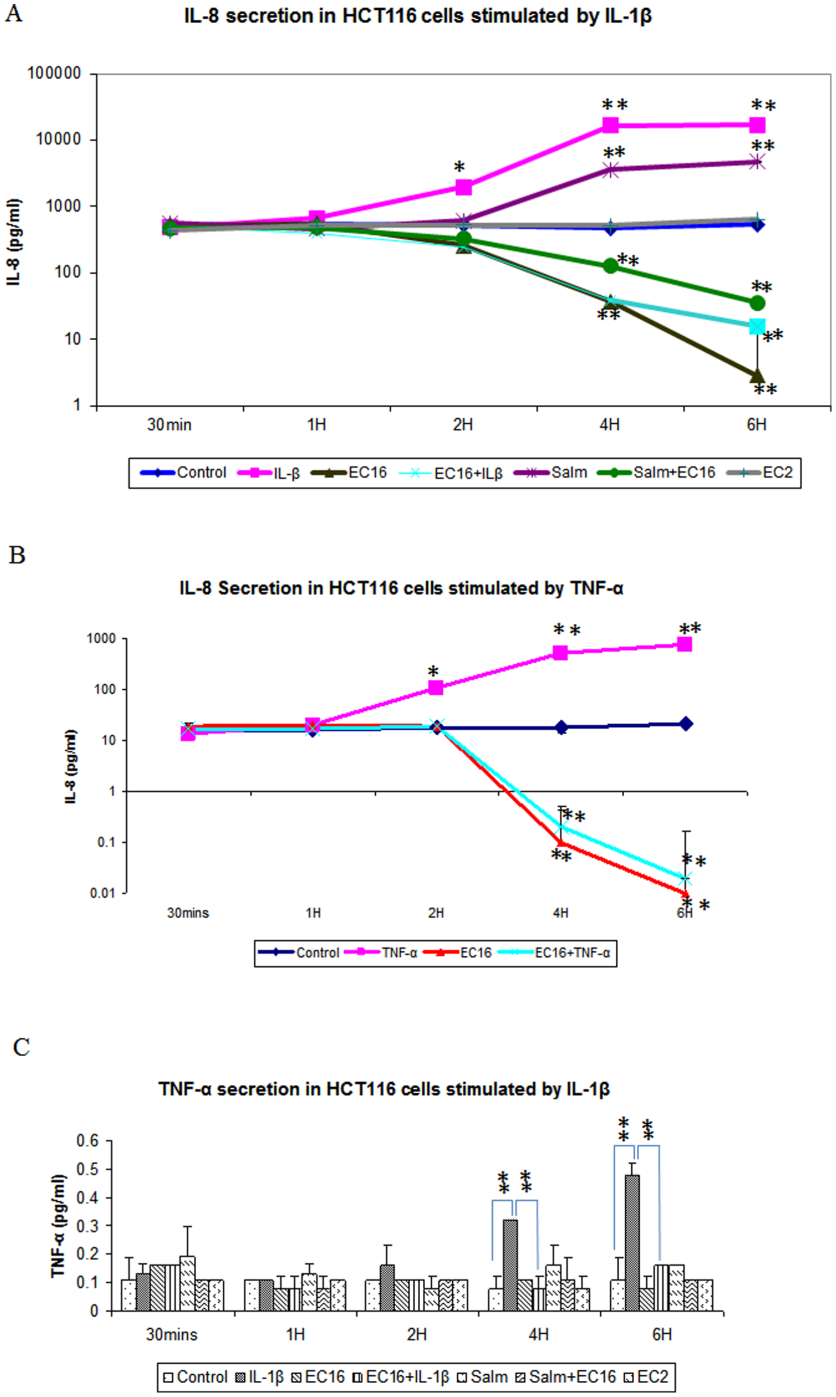
Cytokine secretions in HCT116. IL-8 (A) and TNF-α (C) production in HCT116 with the treatment of 2 ng/ml of IL-1β and 10^7^ cfu/ml of *S. typhimurium* and *E. faecalis* (EC16 and EC2) as described in Materials and Methods. (B) IL-8 production in HCT116 with the treatment of TNF-α and *E. faecalis* EC16 as described in Materials and Methods. Data was expressed as mean value ±SD. Student's T-test was used for statistical analysis as described in Materials and Methods. *p<0.05, **P<0.01.

We next tested the ability of *E. faecalis* to regulate IL-8 production induced by IL-1β (2 ng/ml), TNF-α (200 ng/ml) and *S. typhimurium* (10^7^ CFU/ml) in Caco-2 and HCT116 cells. We found that *E. faecalis* EC16 suppressed IL-8 production induced by IL-1β, TNF-α and *S. typhimurium* after 1 h of incubation. The suppression of IL-8 production inside the cells was observed at 30 mins of treatment (Figure S2A,B). This phenomenon was observed in both HCT116 (Figure 4A,B) and Caco-2 (Figure S2C) cells. Interestingly, *E. faecalis* EC2 could not suppress IL-8 production either inside or outside of IECs, suggesting distinctive ability of these four *E. faecalis* strains (EC1, EC3, EC15, EC16) in regulating IL-8 production.

### *E. faecalis* suppresses TNF-α expression induced by IL-1β

In addition to IL-8, we tested potential TNF-α regulation by *E. faecalis* in HCT116 and Caco-2 cells. TNF-α is an important regulator of epithelial inflammation. TNF-α levels are elevated in both human inflammatory bowel diseases and animal models of intestinal inflammation [28]–[30]. As expected, we found that TNF-α secretion from HCT116 (Figure 4C) and Caco-2 (Figure S2D) cells were activated by IL-1β. This increase secretion, however, was attenuated by *E. faecalis* EC16. Interestingly, ICAM1, IL-2, IL-5, IL-17 and INF-γ induction by IL-1β and *S. typhimurium* was suppressed by EC16 in Caco-2 cells (Figure S2D).

### *E. faecalis* regulates multiple immune-signaling pathways

**Figure 5:**
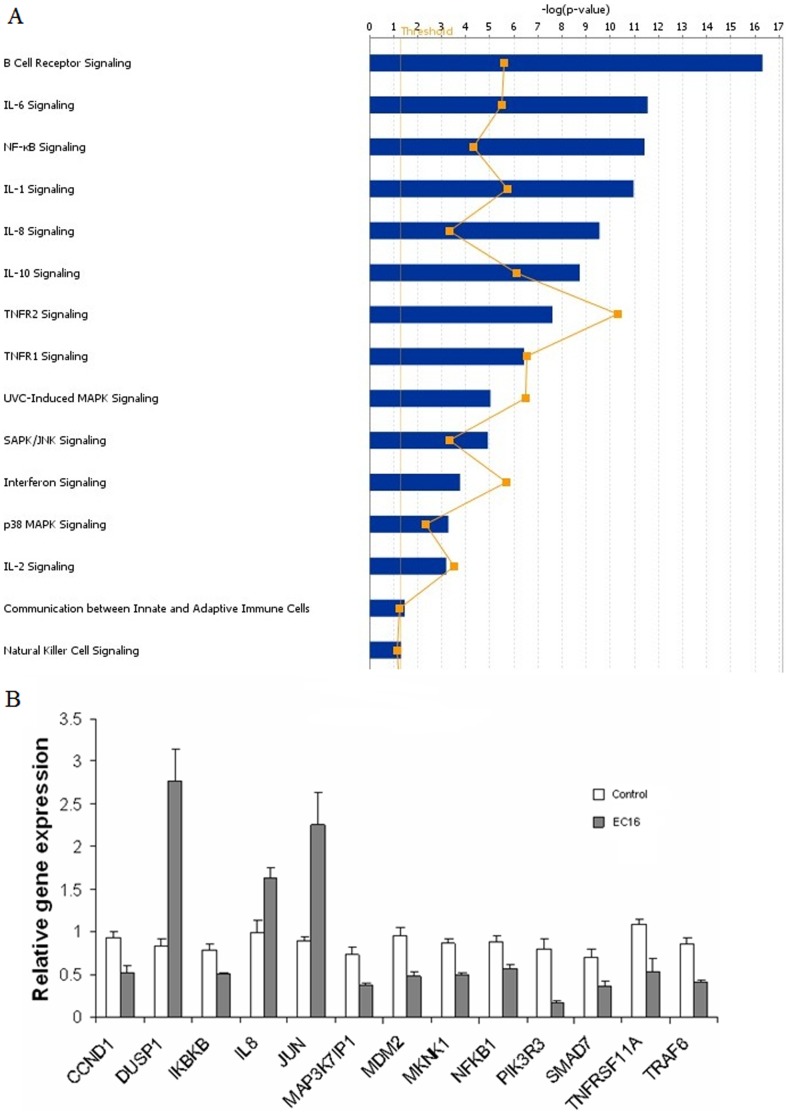
IPA and Realtime PCR analysis. Ingenuity pathway analysis (A) and Real time PCR (B) on *E.faecalis* treated HCT116 cells. All pathways and genes showed in the figure were significantly (p<0.05) regulated in HCT116 cells with the treatment of *E. faecalis* EC16 for 6 h at a MOI of 100. Experiments were done on 3 biological replicates.

Our finding that *E.faecalis* could suppress IL-8 expression led us to investigate if these bacteria could regulate other immune-signaling pathways. We tested immune-gene expression by the four strains of *E. faecalis* (EC1, EC3, EC15, EC16) using cDNA microarray analysis. Potential expression changes for 406 immune-signaling genes were assayed in Caco-2 cells after 6 hour coculture with *E. faecalis* isolates. Acquired data from array membranes were initially scanned (Figure S3A) and volcano plots were obtained to identify statistically significant gene expression changes (Figure S3B). A partial list of the genes that demonstrate statistically significant expression changes (>1.5 fold) by cDNA microarray analysis is provided in Table 2. From the data of microarray, we found several signaling pathways that may be involved in the anti-inflammatory effects of the four *E. faecalis* strains. Using Ingenuity Pathway Analysis, we mainly identified cytokine signaling (IL-1, IL-2, IL-6, IL-8 and IL-10 signaling), SAPK/JNK signaling, P38 MAPK signaling and NF-κB signaling are responsible for the responses (Figure 5A). Together these data suggest that *E. faecalis* may alter multiple immunomodulatory pathways simultaneously.

**Table 2:** Significantly regulated gene (P<0.05) in Caco-2 cells treated with *E. faecalis* for 6h measured by cDNA microarray.

Functional category	Gene population
Response protein	***GBP1,DUSP1***; SLC2A4, NPPB,
Receptors	***EGFR***;IL8RA, TNFRSF11A, P2RX7
Transcription factors	***MLLT7, EGR1, ETS1***;MAPKAPK2, MAPKAPK3, MAX, MEF2B, MKNK1, SP1
AKT& PI3K family	PIK3R3, TCL1A, TCL1B
MAKP family	***MAPK13***;MAPK7, MAP4K1
Adaptor	***TNFAIP3, MDM2;***
Nuclear factors	NCOA1, SPI1
Signal transduction kinases	IKBKB, MAP3K7IP1, PRKCA
Rel/NF-kb	NFKB1, NFKBIL2, NFKB2
Others	***SMAD4, SMAD1, BCL2***; NFATC2, NCK2, PPP3CB, PPP3CC, CTLA4, SMAD6.

Note: Bold italic labeled genes are those upregulated. The rest are downregulated genes.

From the microarray analysis, we selected 46 genes for further study using TLDA. The gene ID and Taqman probes are listed in Table S1. Each of the four *E. faecalis* isolates demonstrated a similar pattern on the immune-gene regulation in Caco-2 and HCT116 cells. Data for gene-regulation by isolate EC16 is provided as a representative example (Figure 5B). To our surprise, we found that IL-8 mRNA was unregulated. We also found that DUSP1, a reported MAPK phosphatase that can attenuate MAPK signaling, was strongly upregulated. Other genes involved in MAPK signaling were downregulated in Caco-2 cells, namely MAP3K7IP1, a positive regulator of the MAP kinase cascade [31]; MKNK1, a target of ERK and activator of CREB-mediated proliferation and differentiation [32] and MAPKAPK2, a target of p38 MAP kinase involved in many cellular processes including inflammatory responses (Figure S4A). Other MAPK family members like MAPK7 in Caco-2 cells, MAPK9 in HCT116 cells were also suppressed by *E. faecalis* (Figure S4B,C). From the above data, we hypothesize that early-colonizing *E. faecalis* might influence the inflammatory responses in the host by regulating MAPK signaling pathway. Furthermore, consistent with observations from microarray, TLDA results also showed that NF-κB1 and IKBKB were suppressed by *E. faecalis* in both Caco-2 and HCT116 cells (Figure S4D,E) suggesting that *E. faecalis* suppressed NF-κB1 signaling pathway at transcriptional level.

### *E. faecalis* suppresses activation of P38, P-JNK and C-JUN

**Figure 6:**
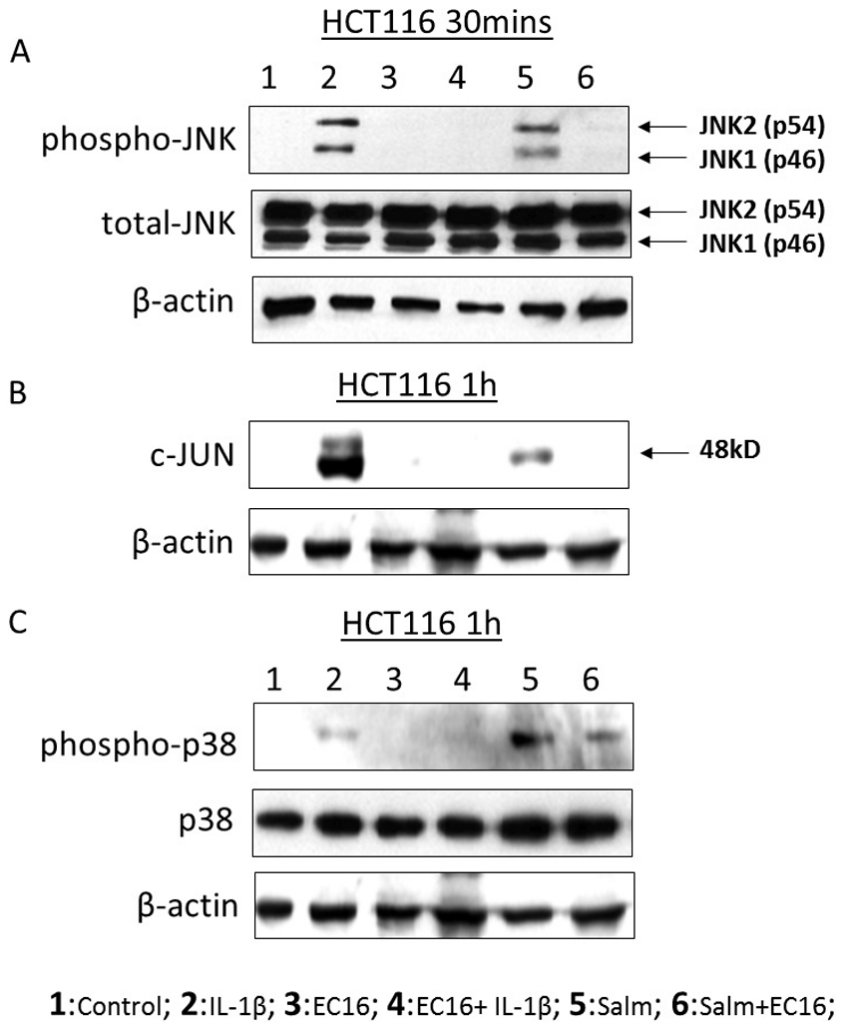
Protein expression in HCT116JNK expression (A) at 30 mins, c-JUN (B) and P38 (C) expression at 1 h in HCT116 with the treatment of 2 ng/ml of IL-1β and 10^7^ cfu/ml of *S. typhimurium, E. faecalis* EC16 as described in Materials and Methods. Cells were then lysed and proteins were tested using western blot. Three independent experiments were done.

Because factors regulating MAPK signaling were altered upon *E.faecalis* exposure, we hypothesized that these bacteria may attenuate IL-8 production, at least in part through inhibiting MAPK pathways. To test this, we examine MAPK expression and phosphorylation by immunoblotting. Whereas p-JNK was strongly and transiently activated by IL-1β and *S. typhimurium* in HCT116 cells within half hour of treatment, *E. faecalis* suppressed this activation (Figure 6A). Consistent with this finding, cJUN which is downstream of JNK, was activated by IL-1β and *S. typhimurium* at one hour while suppressed by *E. faecalis* EC16 (Figure 6B). Similarly, the presence of phosphorylated p38 was increased by incubation with either IL-1β or *S. typhimurium* at one hour, which was abrogated by coculture with *E. faecalis* EC16 (Figure 6C). Therefore, *E. faecalis* can inhibit JNK and p38 signaling as one potential means of suppressing IL-8 production. In contrast, ERK phosphorylation did not change upon the treatment of IL-1β, *S. typhimurium* and *E. faecalis* (Figure S5), suggesting that ERK does not impact IL-8 production in these cells. These data suggest that *E.faecalis* may suppress inflammatory signaling in IECs through reducing the activation of JNK and p38.

### *E.faecalis* suppress IL-1β and TNF-α expression in DSS induced colitis mice model

**Figure 7:**
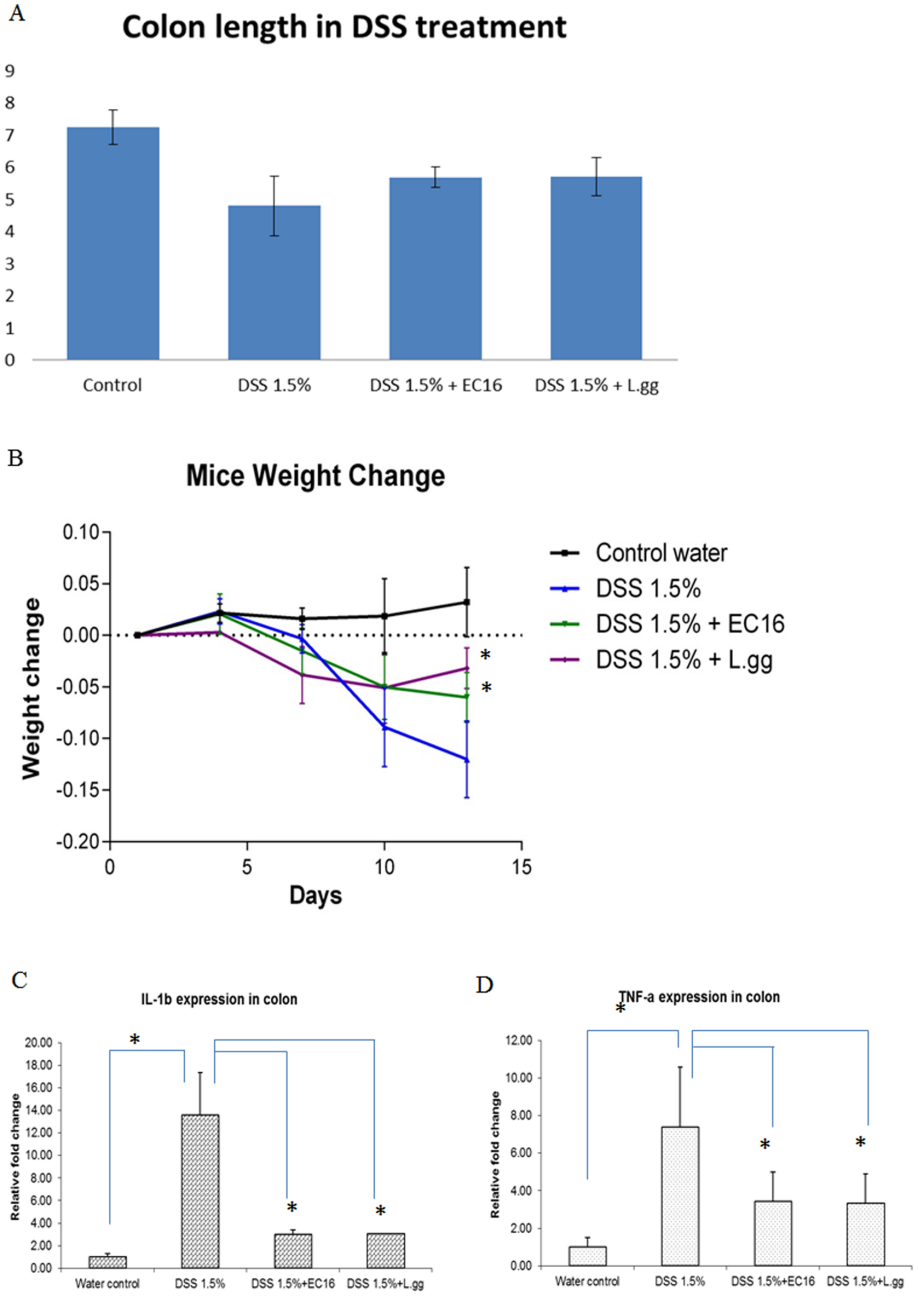
*E.faecalis* treated DSS mild colitis model. Colon lengths (cm) (A) and Mice weight change (B) with 1.5% DSS with or without EC16 and L.GG treatment as described in Material and Method. Real time PCR on IL-1β (C) and TNF-α (D) expression in colon. Each group contains 6–8 mice. Data was expressed as mean value ±SD. Student's T-test was used for statistical analysis as described in Materials and Methods. * p<0.05, ** P<0.01.

To examine the immunomodulatory effects of *E.faecalis in vivo*, we induced colitis using Dextran Sulphate Sodium salt (DSS) in mice and then treated them with *E.faecalis* EC16 or *Lactobacillus rhamnosus GG* (L.gg), a well studied probiotic strain. Colon length, which is an indicator of inflammation, was shortened by DSS treatment. Consistent with an immunorepressive role, both *E.faecalis* and L.gg significantly alleviated this shortening (Figure 7A). Furthermore, *E.faecalis* as well as L.gg prevented DSS-induced weight loss in these animals (Figure 7B). IL-1β and TNF-α which were activated by DSS were significantly down regulated by EC16 and L.gg (Figure 7C,D)

## Discussion

In this study, we describe the isolation and primary characterization of *E.faecalis* strains isolated from healthy newborns in Indonesia. *E.faecalis* represented the most frequently isolated bacteria from the children and selected isolates possessed ability to strongly inhibit inflammatory markers in IECs. The selected isolates with the most potent anti-inflammatory effect were characterized to define a potential mechanism of action and we found that MAPK pathways were regulated. Specifically, *E. faecalis* was able to mitigate activation of JNK and p38 coincident with reduced expression of IL-8 *in vitro* as well as IL-1β and TNF-α *in vivo*.

Enterococci have previously been reported among the common early colonizers in humans [6]. Thus our study population in Indonesia provides a surprising consistency of early colonizing bacteria. Since *E.faecalis* accounts for 90-95% commensal enterococci in adult human intestines, the finding of *E.faecalis* as more prominent than the next most prominent species, *E.faecium*, is not unexpected. In our study *L.casei* was the second most frequently isolated lactic acid bacteria in 3 day old infants. Although the study cohort is not big, to our knowledge this is the first report of *lactobacillus* among early colonizers, which might reflect environmental differences for this Indonesian cohort.

None of the lactobacilli isolates were strongly immunosuppressive as determined by IL-8 expression levels. Certain *E.faecalis* isolates, on the other hand, could strongly inhibit IL-8 in IECs. Our previous findings showed that *E.faecalis* can induce anti-inflammatory cytokine IL-10 in intestinal epithelial through PPAR-gamma [33]. Taken them together, this may explain lower abundance enterococcus colonization [34] and high rate of NEC in preterm infant[35]. Furthermore, infants who developed NEC carry less *E.faecalis* [13], [36]. Therefore, *E.faecalis* may possess the capacity to modulate and attenuate the inflammatory responses further to prevent inflammatory diseases such as NEC in infants. Interestingly, the behavior of the three IEC lines differed substantial in response to many bacteria, suggesting that, in contrast to standard analyses, the use of a single IEC for characterization may be inadequate. The anti-inflammatory effects were confirmed in an *in vivo* system. This finding is consistent with previous study showing that *E.faecalis* has a great protective effect in DSS-induced experimental colitis model in mice [37]. It is not clear, at this stage, why only certain isolates of *E.faecalis* in this study could reduce IL-8 production *in vitro*. It will be interesting to potentially examine these other isolates for their behavior in DSS-induced colitis model *in vivo*.

Interestingly, while *E.faecalis* isolates decreased IL-8 protein level inside and outside the cells, its mRNA level was upregulated. Recent researches also showed that MAPK can regulate eIF4E [38] and eIF4E overexpression is associated with increased IL-8 expression [39]. Therefore, inhibition of MAPK pathway might suppress eIF4E, which results in reduction in IL-8 translation. In the DSS-colitis model as well as patients with inflammatory bowel disease (IBD), p38 levels are increased in the muscularis propria of colonic tissue [20]. When treated with p38 inhibitor, mucosal IL-1β and TNF-α levels were reduced in DSS colitis model [21] consistent with what we found for *E.faecalis* treatment. Thus, the phenomena we see with *E.faecalis* may be exclusively a result of MAPK inhibition. Alternately, *E.faecalis* may be affecting other pathways, for example our previous findings showed LAB can suppress TLR3, TLR9 and TRAF6 mRNA levels [40]. Therefore *E.faecalis* could suppress TLR pathways and further suppress MAPK pathway as well as NFκB-mediated transcription.

*E.faecalis*, as one of the first colonizer, could suppress the inflammatory responses and shape the immune system. Infant intestine usually undergo acute inflammation when exposed to Gram-negative bacteria. The presence of *E. faecalis* may help the intestine maintain the immune balance in response to such challenges. Our finding that *E.faecalis* performed as good as recognized probiotics indicates their potential to serve as a probiotic. However, the therapeutic effects must be examined thoroughly as *E.faecalis* was also reported as an opportunistic pathogen in hospital infections. Since we found dead *E.faecalis* also have the ability to suppress IL-8 secretion, the use of dead *E.faecalis* could mitigate the risk of opportunistic *E.faecalis* infection.

## Materials and Methods

### Bacteria culture and Identification

All bacterial strains were isolated from 16 healthy infants in Indonesia. This study is reviewed and approved by the National University of Singapore Institutional Review Board (NUS IRB), approval no. NUS1469. A written consent form was obtained from participants guardians. The inclusion criteria included natural birth and breast feeding. The exclusion criteria included antibiotic intake 2 weeks before and within the study, received probiotics/culture milk 2 months prior and during the course of the study.

Those bacteria were first Gram stained followed by API 50 CH test strips for rod-shaped bacteria and rapid ID 32 STREP test for the coccal shaped strains. All bacteria strains were then cultured and extracted for DNA. 16S rDNA were directly sequenced using primer 1100 reverse 5V-GGGTTGCGCTCGTTG-3V to obtain partial sequence of the 16S rDNA [41]. Phylogenetic tree calculation was based on a sequence distance method and utilizes the Neighbor Joining (NJ) algorithm of Saitou and Nei [42].

### Cell culture and Infection

Caco-2, HT-29 and HCT116 were obtained from American Type Culture Collection (Manassas, VA) and maintained in ATCC recommended medium. Before cell infection, 1×10^5^ cells were cultured in sterile 24-well flat-bottom plates (Nalge Nunc International, USA) for 24 hours. Caco-2, HT-29 and HCT116 cells were incubated in fresh medium without (control) or with bacteria at a multiplicity of infection (MOI) of 100 for 6 hours. Supernatants were harvested for ELISA assay (BD bioscience, San Diego, CA). and proteins were harvested for Western blotting analysis.

### Conditional Medium and Cell Inserts

The cell culture supernatants obtained from IECs, bacteria and coculture of bacteria and IECs were harvested sterilely and conditional media were then added to the cell cultures prepared. The supernatant were then collected for cytokines assay. For cell inserts test, HCT116 cells were plated in wells of Transwell (USA) multiple well plate. 100 µl of bacterial suspensions were added to each insert. To sonicate bacterial cells, protease inhibitors were added to the suspensions. The bacterial cells were then disrupted by sonication (SANYO, Japan) on ice for 10 cycles with 30 sec pulses and 1min rest [43]. Cell debris was pelleted down and added in the cell culture prepared as above. Another bacterial suspensions were exposed to UV light for 5 min [44] to make sure at least 99% bacteria were killed and then co-culture with cells.

### TNF-α, IL-1β and *S. typhimurium* induced cytokine secretion and protein production

200 ng/ml TNF-α (Preproteck, INC, rocky hill, NJ), 0.2 ng/ml IL-1β (Preproteck, INC, rocky hill, NJ) and *S. typhimurium* at a MOI of 100 were added to Caco-2 and HCT116 cells. Cells were then infected with *E. faecalis* EC16 with or without TNF-α/IL-1β/*S. typhimurium*. Cells without any treatment were used as controls. The cells were then cultured at 37°C with 5% CO_2_ for 30 mins, 1 h, 2 h, 4 h and 6 h. Supernatants were collected and the concentration of IL-8 was determined by ELISA (BD bioscience, San Diego, CA). Other cytokines like INF-γ, TNF-α, IL-2, IL-5, IL-17 and ICAM-1 were determined using cytokine assay (Bio-Rad, USA). The proteins were harvested for Western blotting assay.

### Microarray analysis

A total of 406 human immunology signaling pathway related cDNA clones were detected in this study (Superarray, USA) according to manufacture's instruction. Raw data are available at Gene Expression Omnibus with accession number of GSE56485. Signals were analyzed using the web-based GEArray Expression Analysis Suite (Supperarray, US). GeneSpring GX 7.3.1 and Ingenuity pathway analysis was also used to analyze the data. Data were excluded with bad or absent flags. In this experiment, 2-3 replicates were done for one treatment. Student t test was applied for controlling the false-positive rate (*P*<0.05 was considered significant), clustering analysis was generated by the software.

### TaqMan Low Density Array (TLDA)

The RNA was harvested using the RNA extraction kit (Roche, Switzerland). A 48-well format Taqman low density array was designed for a subset of genes that were differentially expressed in the array experiments including two endogenous controls 18 s and β-actin (Applied Biosystems, USA). Genes and ABI assay IDs are listed in Table S1. 0.5 µg total RNA was converted to cDNA using High-Capacity cDNA archive kit (Applied Biosystems, USA) and 10 ng cDNA in 100 µl TaqMan universal PCR master mix (Applied Biosystems, USA) was used for each port and run on an ABI 7900 system (Applied Biosystems, USA). Data was analyzed using the SDS2.2 software where baseline and threshold settings were automatically adjusted.

### Sodium-Dodecyl Sulfate-Polyacrylamide Gel Electrophoresis (SDS-PAGE) and immuno blotting

Sodium-dodecyle sulfate-polyacrylamide gel electrophoresis was performed on a 10% gel by using 20 µg per lane of whole cell lysate. Electrophoresis was carried out using consistent voltage of 75 volts for approximately one and a half hour and then transferred onto a 0.22 µm Nitrocellulose membrane (Biorad, USA) at 85 volts for 2 hours in cold room. The membrane was then blocked in Tris buffer saline-Tween (TBST) containing 5% skim milk for at least 1 h. Specific primary antibodies were then diluted and the membrane was incubated overnight at 4 °C on an orbital shaker (Bellco, USA). After washing, appropriate horseradish peroxidase (HRP)-conjugated secondary antibodies diluted in TBST containing 5% skim milk were added into the membrane and incubated for 1 hour at room temperature on an orbital shaker (Bellco, USA). Visualization of the immunolabeled bands was then carried out using ECL Plus Western Blotting Detection Reagents or ECL Advance Western Blotting Detection Kit (GE Healthcare, UK) according to manufacturer's instruction. The signals were exposed on X-ray film (Koda, USA).

### Animals

Male C57BL/6 mice (8 to 10 weeks of age) were kept in SingHealth Experimental Medicine Centre and housed in collective cages at 22±1°C under a 12-h light/dark cycle (lights on at 07:00 h) with free access to laboratory chow and autoclaved tap water. Experiments were performed during the light phase of the cycle. The experimental procedures were previously approved by SingHealth Institutional Animal Care and Use Committee (IACUC) on the Ethical Use of Animals, where the study was carried out, and were conducted in accordance with Singapore regulations on animal welfare.

### DSS induced mild colitis model in mice

Male C57BL/6 mice (*n* = 6–8 per group) were provided with a solution of filtered water containing 1.5% dextran sodium sulfate (DSS) (TdB Consultancy AB, Uppsala, Sweden) over a 12-day period. Every other day, the total of 200 ml of 1.5% DSS solution was replenished. The total volume of DSS solution consumed per mouse was proximally 4.4 ml/day. No differences between experimental groups were observed. On the third day of 1.5% DSS treatment, treatment group were fed with 10∧7 CFU/100 ml EC16 or 10∧7 CFU/100 ml L.GG using a gavage needle respectively. Control group were fed with PBS. The animals were provided with 1.5% DSS till day 12. On day 12, the animals were euthanized, colon were removed for length measurement and frozen in liquid nitrogen for future analysis.

### Statistical analysis

Data are expressed as means ± SD. Significance of differences was determined using the Student's T-test and analysis of variance. P values<0.05 were considered to be statistically significant.

## Supporting Information

Figure S1 **IL-8 secretions in Caco-2 (A), HT-29 (B) and HCT116 (C) with the treatment of** ***Lactobacillus*** **and apoptosis assay in HCT116 (D,E).** A total number of 10^7^ cfu/ml bacteria were added into the cells for 6 h. Supernatants were harvested for cytokine assay as described in Materials and Methods. Three independent experiments were compiled to produce the data shown. Data were expressed as mean value ±SD. D. Apoptosis assay in HCT116 control. E. Apoptosis assay in HCT116 with EC16 treatment. One representative assay was shown from three independent experiments.
(TIF)

Figure S2 **Cytokine productions in IECs.** IL-8 production inside HCT116 cells (A, B), IL-8 secretion (C) and ICAM1, IL-2, IL-5, IL-17, IFN-γ and TNF-α in Caco-2 cells (D) with the treatment of 2 ng/ml of IL-1β and 10^7^ cfu/ml of *S. typhimurium* and *E. faecalis* (EC16 and EC2) as described in Materials and Methods. Three replicates were done. Data was expressed as mean value ±SD.
(TIF)

Figure S3 **Gene expression in Caco-2 cells tested using cDNA microarray.** (A) The original photo of EC16 after exposure. (B) Volcano plot of EC16 obtained using Gene Spring software. Red dots represent the significantly changed genes. Yellow dots represent the genes not be regulated significantly. Only one of five sets of EC16 data were used as a representation.
(TIF)

Figure S4 **Real-time PCR on** ***E.faecalis*** **treated HCT116 (dot) and Caco2 (black) cells.** Experiments were done on 3-4 biological replicates and 2 technical replicates. Folds changes >1.5 and T-test with a P<0.05 was considered significant change.
(TIF)

Figure S5 **ERK expression in HCT116 cells at 30 mins with the treatment of 2 ng/ml of IL-1β and S. typhimurium with and without E. faecalis EC16 at a MOI of 100.** Total protein was harvested and protein production was analyzed using Western blotting as described in Materials and Methods. Experiments were repeated three times.
(TIF)

Table S1 **Gene ID and Taqman Primers ID.**
(DOCX)
