# Supplementary figures and images for: Enterococcus faecalis from Healthy Infants Modulates Inflammation through MAPK Signaling Pathways
Source: PLoS One. 2014 May 15;9(5):e97523. doi: 10.1371/journal.pone.0097523 (PMC4022717; doi:10.1371/journal.pone.0097523)

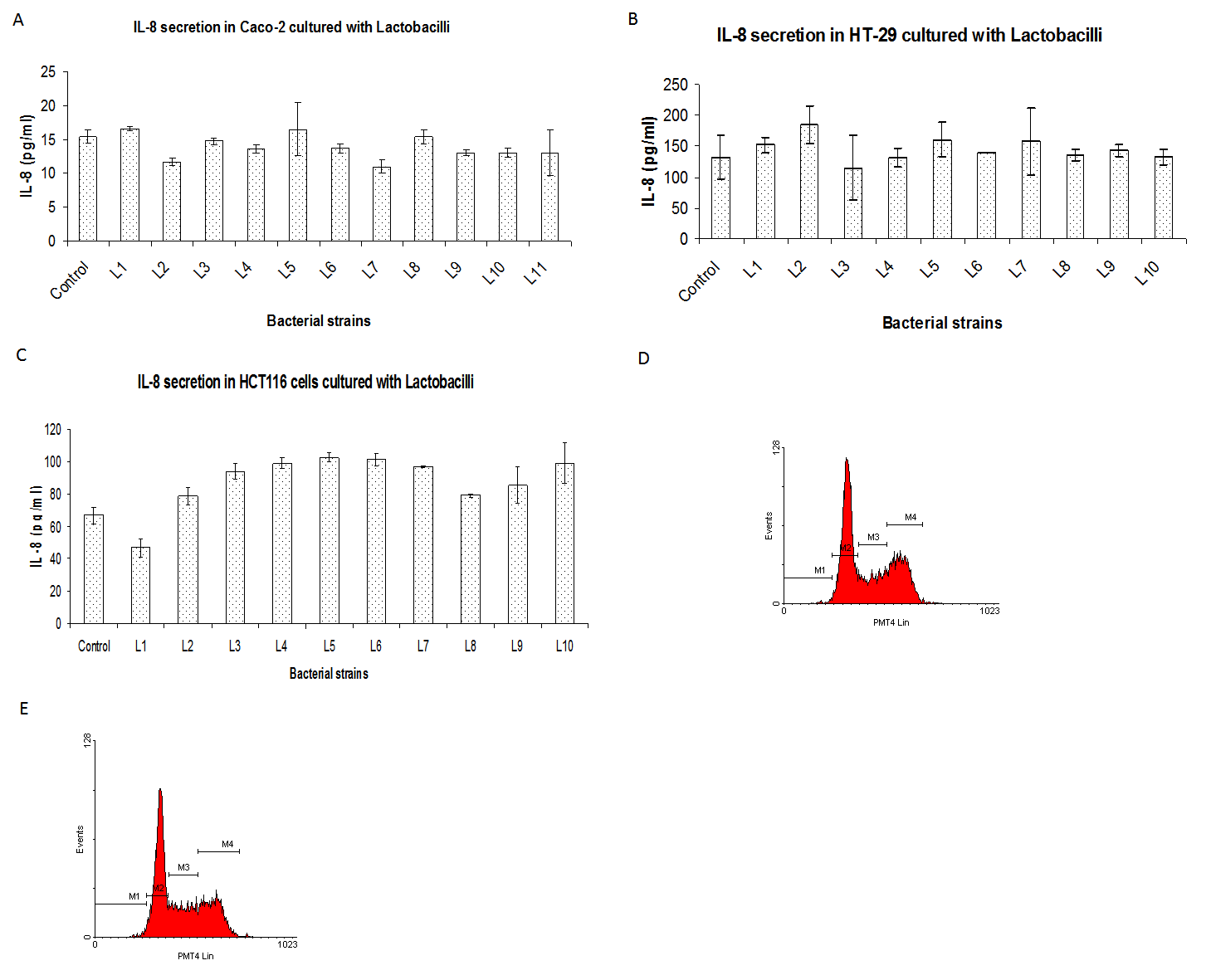

Supplement: Figure S1 — IL-8 secretions in Caco-2 (A), HT-29 (B) and HCT116 (C) with the treatment of Lactobacillus and apoptosis assay in HCT116 (D,E). A total number of 107 cfu/ml bacteria were added into the cells for 6 h. Supernatants were harvested for cytokine assay as described in Materials and Methods. Three independent experiments were compiled to produce the data shown. Data were expressed as mean value ±SD. D. Apoptosis assay in HCT116 control. E. Apoptosis assay in HCT116 with EC16 treatment. One representative assay was shown from three independent experiments. (TIF) [file pone.0097523.s001.tif]

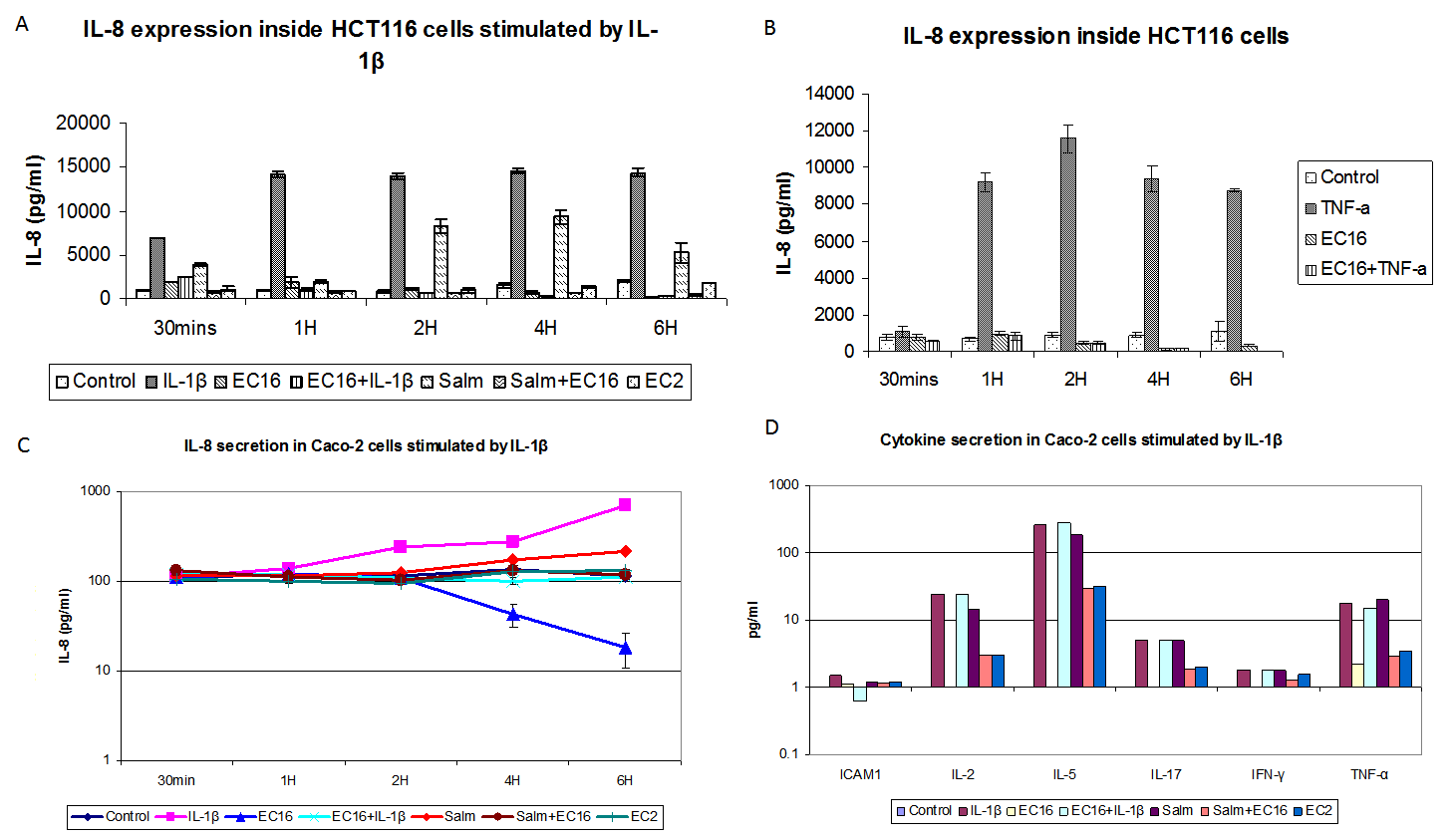

Supplement: Figure S2 — Cytokine productions in IECs. IL-8 production inside HCT116 cells (A, B), IL-8 secretion (C) and ICAM1, IL-2, IL-5, IL-17, IFN-γ and TNF-α in Caco-2 cells (D) with the treatment of 2 ng/ml of IL-1β and 107 cfu/ml of S. typhimurium and E. faecalis (EC16 and EC2) as described in Materials and Methods. Three replicates were done. Data was expressed as mean value ±SD. (TIF) [file pone.0097523.s002.tif]

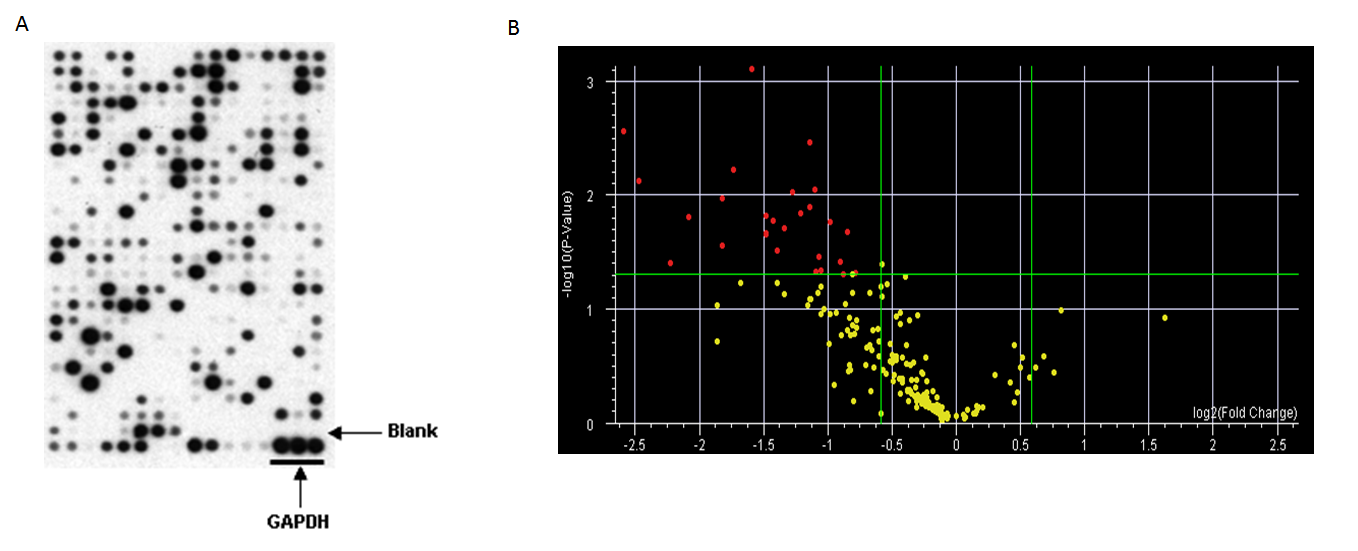

Supplement: Figure S3 — Gene expression in Caco-2 cells tested using cDNA microarray. (A) The original photo of EC16 after exposure. (B) Volcano plot of EC16 obtained using Gene Spring software. Red dots represent the significantly changed genes. Yellow dots represent the genes not be regulated significantly. Only one of five sets of EC16 data were used as a representation. (TIF) [file pone.0097523.s003.tif]

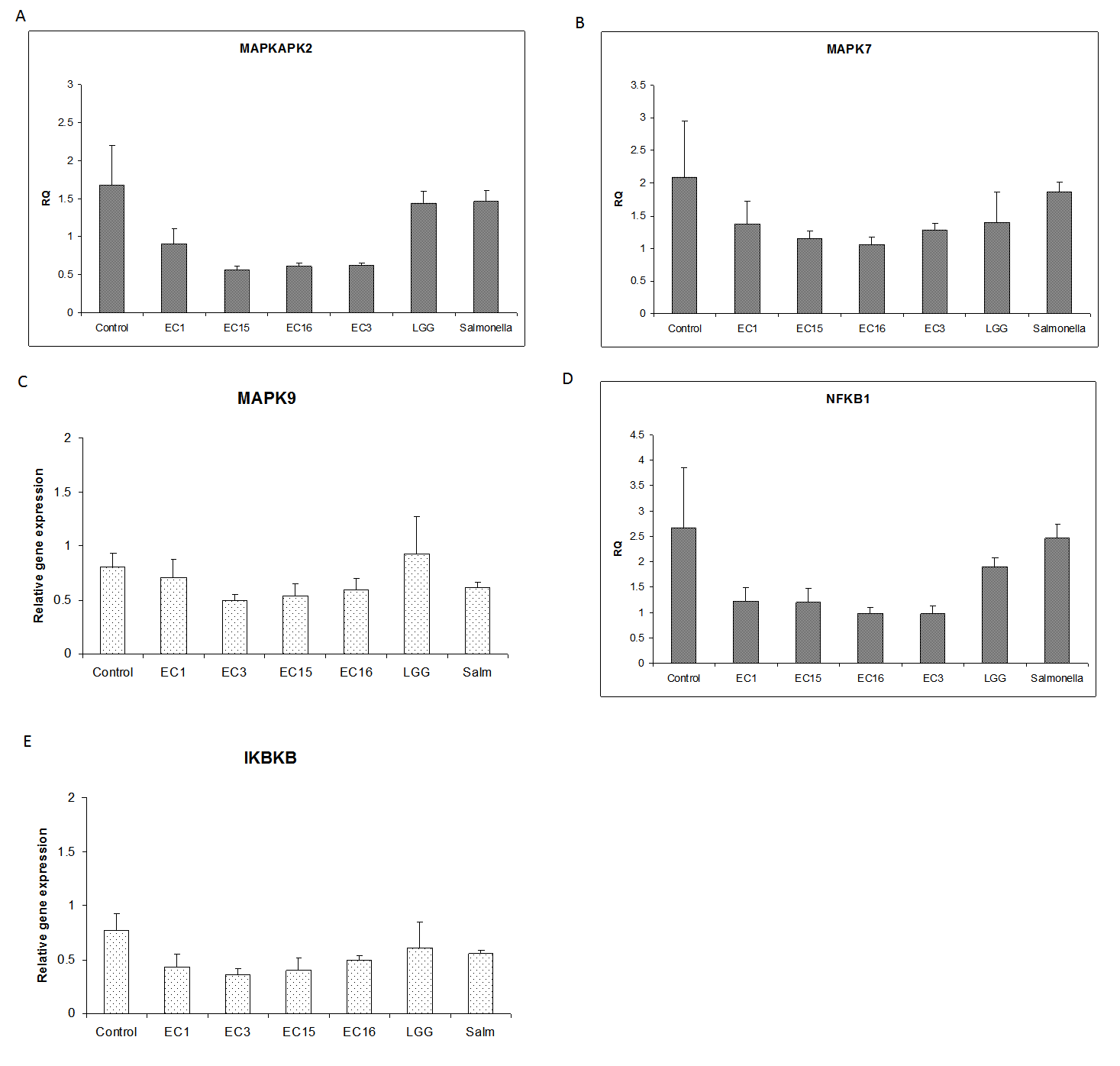

Supplement: Figure S4 — Real-time PCR on E.faecalis treated HCT116 (dot) and Caco2 (black) cells. Experiments were done on 3-4 biological replicates and 2 technical replicates. Folds changes >1.5 and T-test with a P<0.05 was considered significant change. (TIF) [file pone.0097523.s004.tif]

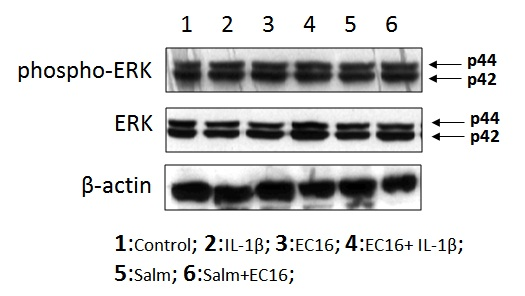

Supplement: Figure S5 — ERK expression in HCT116 cells at 30 mins with the treatment of 2 ng/ml of IL-1β and S. typhimurium with and without E. faecalis EC16 at a MOI of 100. Total protein was harvested and protein production was analyzed using Western blotting as described in Materials and Methods. Experiments were repeated three times. (TIF) [file pone.0097523.s005.tif]
