# Supplementary material for: Enterococcus faecalis from Healthy Infants Modulates Inflammation through MAPK Signaling Pathways
Source: PLoS One. 2014 May 15;9(5):e97523. doi: 10.1371/journal.pone.0097523 (PMC4022717; doi:10.1371/journal.pone.0097523)
Supplement: Table S1 — Gene ID and Taqman Primers ID. (DOCX) [file pone.0097523.s006.docx]

# Table S1 Gene ID and Taqman Primers ID

| **GeneBank ID** | **Human Gene ID** | **Gene symbol** | **Assay ID** |
| --- | --- | --- | --- |
| NM_053056 | Hs.523852 | CCND1 | Hs00277039_m1 |
| NM_000077 | Hs.512599 | CDKN2A | Hs00233365_m1 |
| NM_004379 | Hs.516646 | CREB1 | Hs00231713_m1 |
| NM_004417 | Hs.171695 | DUSP1 | Hs00610256_g1 |
| NM_005225 | Hs.96055 | E2F1 | Hs00153451_m1 |
| NM_005252 | Hs.25647 | FOS | Hs00170630_m1 |
| NM_000874 | Hs.549042 | IFNAR2 | Hs00174198_m1 |
| NM_001556 | Hs.413513 | IKBKB | Hs00233284_m1 |
| NM_000572 | Hs.193717 | IL10 | Hs00174086_m1 |
| NM_014432 | Hs.445868 | IL20RA | Hs00205346_m1 |
| NM_000589 | Hs.73917 | IL4 | Hs00174122_m1 |
| NM_000584 | Hs.624 | IL8 | Hs00174103_m1 |
| NM_000634 | Hs.194778 | IL8RA | Hs00174146_m1 |
| NM_002228 | Hs.525704 | JUN | Hs00277190_s1 |
| NM_006116 | Hs.507681 | MAP3K7IP1 | Hs00196143_m1 |
| NM_005204 | Hs.432453 | MAP3K8 | Hs00178297_m1 |
| NM_002751 | Hs.57732 | MAPK11 | Hs00177101_m1 |
| NM_002754 | Hs.178695 | MAPK13 | Hs00234085_m1 |
| NM_002749 | Hs.150136 | MAPK7 | Hs00611114_g1 |
| NM_002752 | Hs.484371 | MAPK9 | Hs00177102_m1 |
| NM_004759 | Hs.519276 | MAPKAPK2 | Hs00358962_m1 |
| NM_002392 | Hs.369849 | MDM2 | Hs00242813_m1 |
| NM_005919 | Hs.153629 | MEF2B | Hs00232232_m1 |
| NM_003684 | Hs.371594 | MKNK1 | Hs00374376_m1 |
| NM_005373 | Hs.82906 | MPL | Hs00180489_m1 |
| NM_172390 | Hs.534074 | NFATC1 | Hs00232342_m1 |
| NM_003998 | Hs.431926 | NFKB1 | Hs00231653_m1 |
| NM_002502 | Hs.73090 | NFKB2 | Hs00174517_m1 |
| NM_003629 | Hs.170510 | PIK3R3 | Hs00177524_m1 |
| NM_006221 | Hs.465849 | PIN1 | Hs00749260_s1 |
| NM_006244 | Hs.75199 | PPP2R5B | Hs00196561_m1 |
| NM_005903 | Hs.167700 | SMAD5 | Hs00195437_m1 |
| NM_005585 | Hs.153863 | SMAD6 | Hs00178579_m1 |
| NM_005904 | Hs.465087 | SMAD7 | Hs00178696_m1 |
| NM_005905 | Hs.528630 | SMAD9 | Hs00195441_m1 |
| NM_138473 | Hs.524461 | SP1 | Hs00412720_m1 |
| NM_003120 | Hs.502511 | SPI1 | Hs00231368_m1 |
| NM_000660 | Hs.1103 | TGFB1 | Hs99999918_m1 |
| NM_003264 | Hs.519033 | TLR2 | Hs00610101_m1 |
| NM_003265 | Hs.29499 | TLR3 | Hs00152933_m1 |
| NM_003266 | Hs.174312 | TLR4 | Hs00152939_m1 |
| NM_017442 | Hs.87968 | TLR9 | Hs00152973_m1 |
| NM_000594 | Hs.241570 | TNF | Hs00174128_m1 |
| NM_003839 | Hs.204044 | TNFRSF11A | Hs00187192_m1 |
| NM_019009 |  | TOLLIP | Hs00184085_m1 |
| NM_004620 | Hs.444172 | TRAF6 | Hs00371508_m1 |
| NM_001101 |  | ACTB | Hs99999903_m1 |
|  |  | 18S | Hs99999901_s1 |
